# Associations between pre-infection serum vitamin D concentrations and Omicron COVID-19 incidence, severity and reoccurrence in elderly individuals

**DOI:** 10.1017/S1368980024001873

**Published:** 2024-10-07

**Authors:** Jiangjie Chen, Fangying Lu, Bo Shen, Hongfang Xu, Yijun Chen, Qi Hu, Anpeng Xu, Tao-Hsin Tung, Dun Hong

**Affiliations:** 1 Bone Metabolism and Development Research Center, Taizhou Hospital Affiliated to Wenzhou Medical University, Linhai, Zhejiang Province 317000, People’s Republic of China; 2 Department of Orthopedics, Taizhou Hospital of Zhejiang Province Affiliated to Wenzhou Medical University, Linhai, People’s Republic of China; 3 Department of Clinical Laboratory, Taizhou Hospital Affiliated to Wenzhou Medical University, Linhai, People’s Republic of China; 4 Health Management Center, Taizhou Hospital Affiliated to Wenzhou Medical University, Linhai, People’s Republic of China; 5 Department of Orthopedics, Linhai Second People’s Hospital of Taizhou, Linhai, Zhejiang, China; 6 Department of Clinical Research, Enze Medical Center, Taizhou, China

**Keywords:** Vitamin D deficiency, Omicron COVID-19, Vitamin D, Severity of illness, Incidence and reoccurrence rate

## Abstract

**Objective:**

Previous studies suggest a link between vitamin D status and COVID-19 susceptibility in hospitalised patients. This study aimed to investigate whether vitamin D concentrations in elderly individuals were associated with their susceptibility to Omicron COVID-19 incidence, the severity of the disease and the likelihood of reoccurrence during the era of the post-‘zero-COVID-19’ policies in China.

**Design:**

In this retrospective study, participants were categorised into three groups based on their 25(OH)D concentrations: deficiency (< 20 ng/ml), insufficiency (20 to < 30 ng/ml) and sufficiency (≥ 30 ng/ml). The demographic and clinical characteristics, comorbidities and the incidence rate, reoccurrence rate and severity of Omicron COVID-19 were retrospectively recorded and analysed by using hospital information system data and an online questionnaire survey.

**Setting:**

China.

**Participants:**

222 participants aged 60 years or older from a health management centre.

**Results:**

Our findings revealed significant differences in the incidence (*P* = 0·03) and recurrent rate (*P* = 0·02) of Omicron COVID-19 among the three groups. Participants with lower 25(OH)D concentrations (< 20 ng/ml) exhibited higher rates of initial incidence and reoccurrence and a greater percentage of severe and critical cases. Conversely, individuals with 25(OH)D concentrations ≥ 30 ng/ml had a higher percentage of mild cases (*P* = 0·003). Binary and ordinal logistic regression models indicated that vitamin D supplementation was not a significant risk factor for COVID-19 outcomes.

**Conclusions:**

In the elderly population, pre-infection vitamin D deficiency was associated with increased susceptibility to incidence, severity of illness and reoccurrence rates of Omicron COVID-19.

Vitamin D deficiency and insufficiency constitute a global health problem^(1)^. Subclinical vitamin D deficiency, characterised by low serum concentrations of 25(OH)D, the primary form of vitamin D in the bloodstream, is prevalent in both developed and developing nations^(2)^. In the USA, the prevalence of vitamin D insufficiency stands at 40·9 %, while the prevalence of severe and moderate vitamin D deficiency stands at 2·6 % and 22·0 %, respectively. A higher rate of severe and moderate vitamin D deficiency was observed among non-Hispanic black Americans^(3)^. While vitamin D plays a critical role in regulating Ca and P balance and maintaining bone health, its deficiency has also been linked to heightened inflammation, autoimmune disorders and dysregulation of the immune system across various chronic diseases^(4,5)^.

The emergence of the COVID-19 pandemic in 2019 has ignited considerable debate about the potential role of vitamin D in both preventing and treating COVID-19. Numerous studies have reported a strong association between lower vitamin D levels (serum 25(OH)D) and unfavourable COVID-19 outcomes and prognosis^(6–13)^. However, some studies have failed to establish a definitive connection between vitamin D status and COVID-19 severity and mortality^(14–16)^.

The inconsistencies in findings regarding the impact of vitamin D on COVID-19 severity may stem from methodological variations. In most studies, blood samples were collected from COVID-19 patients upon hospital admission to measure vitamin D concentrations. Subsequently, COVID-19 patients were categorised based on the severity of their illness, and vitamin D levels were compared to determine whether severe COVID-19 cases were more likely to exhibit vitamin D deficiency^(6,17–24)^. However, it is important to note that vitamin D concentrations in these studies were measured after patients had contracted the virus, and the SARS-CoV-2 infection itself might directly influence vitamin D levels^(25)^.

In some studies, vitamin D concentration was determined before SARS-CoV-2 infection. For instance, vitamin D levels were analysed in relation to the severity of COVID-19 in 348 598 patients from the UK Biobank^(26)^. Additionally, a Mendelian randomisation study involving 443 734 individuals included 401 460 participants from the UK Biobank^(27)^. Several meta-analyses have also incorporated a substantial number of cases from the UK Biobank, comprising a significant portion of the analysed patient data^(28,29)^. Another large retrospective study encompassed a cohort of over 190 000 US COVID-19 patients, with vitamin D concentrations obtained from the preceding 12 months^(11)^. Nonetheless, the vitamin D measurements in these aforementioned studies were taken well before SARS-CoV-2 infection, potentially not serving as the most accurate indicators of pre-infection vitamin D status.

An ideal approach would involve assessing vitamin D levels in individuals immediately prior to SARS-CoV-2 infection and subsequently examining the clinical severity of COVID-19 and its outcomes in these patients. However, obtaining blood samples immediately before infection was virtually impractical during the pandemic, as infections often occurred unexpectedly. On 7 December 2022, the Chinese Center for Disease Control and Prevention modified its epidemic prevention policy, relaxing the stringent ‘zero-COVID’ policy that had been in place for nearly 3 years^(30)^. Subsequently, a major outbreak of Omicron infections transpired in late December and January 2023. The peak of positive cases was observed on December 22, with a gradual decline throughout late January 2023^(31)^. Hospitalisations due to COVID-19 reached their zenith on January 5, while the number of deaths peaked on January 4 and subsequently declined by 89·9 % by 30 January 2023^(32)^. Remarkably, in Henan Province, home to nearly 100 million people, the provincial government reported on 9 January 2023 that a staggering 89 % of the provincial population had been infected^(30)^.

In this study, we conducted a retrospective analysis of pre-infection serum vitamin D data obtained from elderly participants (aged 60 and above) within a health management centre. These individuals were screened within 3 months prior to the onset of their illness. Our analysis sought to explore the associations between vitamin D concentrations and the incidence rate, reoccurrence rate and severity of Omicron COVID-19 within this cohort.

## Methods

### Study design and participants

**Fig. 1:**
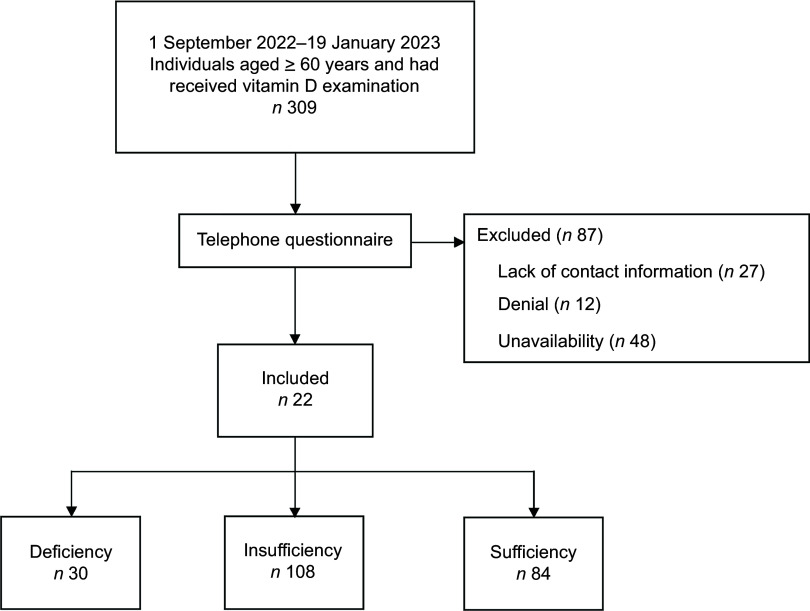
Flow chart diagram for selection of participants

In this retrospective study, we initially identified 309 individuals aged 60 years or older who underwent vitamin D testing within 3 months before contracting COVID-19 between 19 September 2022 and 19 January 2023 from the database of the Physical Examination Center of a large tertiary hospital in Taizhou of Zhejiang Province. We conducted a telephone questionnaire survey, obtained verbal informed consent and assessed the severity of symptoms during and after COVID-19 occurred. And the ethical approval had been approved (Fig. 1).

### Measurement of the serum vitamin D concentrations

Serum concentrations of 25(OH)D, 25(OH)D_2_ and 25(OH)D_3_ were determined using liquid chromatography-tandem MS (AB Sciex QTrap® 3200 Tandem Mass Spectrometer), which was referred to as the ‘gold standard’ method for measuring vitamin D status in human samples^(33–35)^. The total 25(OH)D concentrations were calculated as the sum of 25(OH)D_2_ and 25(OH)D_3_. Participants were categorised into vitamin D deficiency (< 20 ng/ml), insufficiency (20 ng/ml to < 30 ng/ml) and sufficiency (≥ 30 ng/ml) groups, following established criteria^(36)^.

### Timeline of main events in the method

**Fig. 2:**
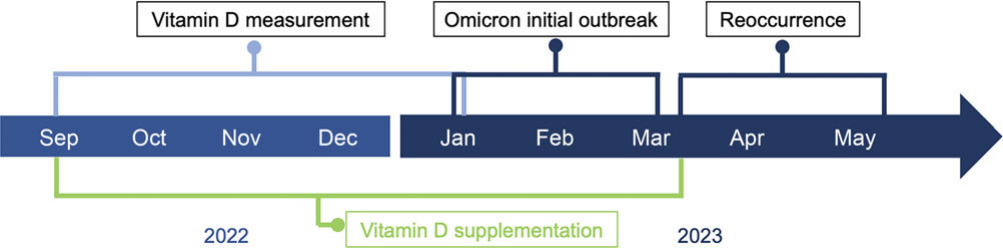
Timeline of the main events in the method

From 19 September 2022 to 19 January 2023, all COVID-19-negative participants underwent vitamin D measurement. From mid-January 2023 to mid-March 2023, the first to the last initial COVID-19 cases occurred. From the beginning of April 2023 to mid-June 2023, the first to the last recurrent COVID-19 case occurred. Continuous use of vitamin D was defined as more than 3months before the end of the initial COVID-19 cases (mid-March) (Fig. 2).

### Definition of smoking status and vitamin D supplement use

Participants who had a history of smoking and continued to smoke were classified as cigarette smokers. Those who regularly consumed vitamin D supplements for more than 3 months were considered vitamin D supplement users.

### Definition of COVID-19-related comorbidities

Chronic obstructive pulmonary disease, hypertension, diabetes, chronic kidney disease and hyperlipidaemia were defined as individuals with past medical history or correspond to ICD10-CM codes (chronic obstructive pulmonary disease: J44·901; hypertension: I10.X02; diabetes: E11; chronic kidney disease: N18·905; hyperlipidaemia: E78·501). CVD was defined as participants who had a history of cardiovascular or cardiac disease. The cerebrovascular disease was defined as participants who had suffered a stroke and brain infarction. The others were referred to as asthma, bronchiectasis, pulmonary tuberculosis, cancer and immunodeficiency diseases.

### Classification of COVID-19 severity

Based on the Diagnosis and Treatment Protocol for COVID-19 Patients (Tentative 9th Edition)^(37)^, the severity of COVID-19 was categorised into level 0 to level 4. Among them, levels 0–1 are mild cases, level 2 belongs to moderate cases, level 3 is severe cases and level 4 is critical cases. Level 0: asymptomatic patients; level 1: the clinical symptoms are mild, including fever ≤ 38°C, fatigue, anosmia and ageusia lasting for less than 7 d, and there is no evidence of pneumonia in chest radiology; level 2: patients have fever > 38°C at least 3 d and respiratory symptoms lasting from 7–14 d. Chest radiology suggests pneumonia; level 3: patients meeting any of the following: rapid progression of clinical symptoms, with > 50 % progression in the lung lesions in chest radiology or with unstable blood pressure or with SpO_2_ < 93 % on room air in resting status or required oxygen uptake or hormone therapy; level 4: severe respiratory failure requiring mechanical ventilation or shock or admission to the intensive care unit or death.

### Definition of COVID-19 and reoccurrence cases

COVID-19 illness was identified based on COVID-19 nucleic acid self-testing kits, positive laboratory tests (PCR or antigen tests), positive antibody results or a positive COVID-19 diagnosis (corresponding to U07·1 ICD10-CM code). Reoccurrence was defined as individuals recovering from a previous infection and contracting COVID-19 again within 3 months using the same testing methods.

### Data collection

Demographic information, including age, gender, BMI, bone mineral density, laboratory parameters and COVID-19-related comorbidities, was collected from the Physical Examination Center database and confirmed in the hospital information system. Three months after the onset of COVID-19, a follow-up telephone survey was conducted by the Physical Examination Center to collect information on smoking status and vitamin D supplementation. Additionally, the severity of COVID-19 disease was assessed based on predefined criteria. Primary parameters included COVID-19 occurrent details, general symptoms, duration of infection, vaccination status, maximum fever temperature, fever duration, SpO_2_ levels, oxygen supplementation, mechanical ventilation, hormone therapy, pneumonia imaging, blood pressure stability and persistent symptoms. Vaccination rates, hospitalisation rates, intensive care unit admissions, deaths and reoccurrence rates within 3 months were also documented.

### Statistics

Continuous variables were presented as mean (sd) and compared using ANOVA. Discrete variables were expressed as numbers (percentages) and compared between groups using Pearson’s *χ*^2^ test and Fisher’s exact test. Binary logistic regression was employed to identify factors associated with COVID-19 incidence and reoccurrence rates. Ordinal logistic regression was used to investigate the association between vitamin D supplementation and the severity of COVID-19. All analyses were conducted using IBM SPSS Statistics software (version 26·0), and significance was defined as *P* < 0·05.

## Results

### Comparison of clinical characteristics among different vitamin D groups

Of the initial 309 subjects, eighty-seven individuals were excluded due to denial (*n* 12), unavailability (*n* 48) or lack of contact information (*n* 27) during the telephone follow-up (Fig. 1). Thus, the final study cohort consisted of 222 participants. The mean age was 68 ± 6 years (range, 61–89 years), with 58 % being female. Participants were categorised into vitamin D deficiency (*n* 30), insufficiency (*n* 108) and sufficiency (*n* 84) groups. There were no significant differences among the three groups in terms of age, gender, BMI, bone mineral density, smoking status or major COVID-19-related comorbidities, including chronic obstructive pulmonary disease, hypertension, diabetes, CVD, chronic kidney disease, cerebrovascular disease, hyperlipidaemia and others (all *P* > 0·05). However, a higher proportion of participants in the deficiency and insufficiency groups reported taking vitamin D supplements (*P* < 0·01). The proportion of vaccination rates among the three groups was relatively high: 99 % of individuals in the vitamin D insufficiency group claimed to have been vaccinated, with a higher vaccination rate compared with the deficiency group (97 %) and sufficiency group (96 %), and there was no significant difference in vaccination rate among the groups (*P* = 0·41). Serum concentrations of 25(OH)D and 25(OH)D_3_ differed significantly among the three groups (*P* < 0·01), while other laboratory parameters showed no significant differences (all *P* > 0·05). (Table 1)

**Table 1:** Comparison of general characteristics in participants with different serum vitamin D concentrations

	Deficiency (*n* 30)		Insufficiency (*n* 108)		Sufficiency (*n* 84)		*P*-value^†^
	Mean	sd	Mean	sd	Mean	sd	
Female							
* n*	20		64		44		0·24
%	67		59		52		
Age (years)	68·83	6·45	68·02	5·59	67·92	5·69	0·74^‡^
BMI (kg/m^2^)	25·38	4·14	24·60	3·12	24·18	2·59	0·19^‡^
BMD	−1·60	0·70	−1·56	1·14	−1·31	1·34	0·36^§^
	*n*	%	*n*	%	*n*	%	
Smoking status	7	23	16	15	12	14	0·48^‡^
Vit-D supplement	12	40	39	36	2	2·4	< 0·001^‡^
Comorbidities							
COPD	7	23	21	19	15	18	0·82
Hypertension	19	63	61	56	47	56	0·79
Diabetes	5	17	12	11	17	20	0·20
CVD	5	17	13	12	7	8·3	0·48
CKD	2	6·7	8	7·4	6	7·1	1·00
Cerebrovascular disease	4	13	7	6·5	4	4·8	0·38
Hyperlipidaemia	18	60	55	51	42	50	0·66
Others	3	10	9	8·3	7	8·3	1·00
Vaccination	29	97	107	99	81	96	0·41
	Mean	sd	Mean	sd	Mean	sd	
Laboratory parameters							
WBC (X10^9^/L)	6·07	1·29	5·97	1·42	6·10	1·60	0·93^§^
NEUT (X10^9^/L)	3·53	0·97	3·60	1·13	3·63	1·18	0·54^§^
LYMP (X10^9^/L)	1·92	0·55	1·82·	0·55	1·96	0·68	0·26^§^
MONO (X10^9^/L)	0·36	0·11	0·35	0·11	0·36	0·11	0·71^§^
EO (X10^9^/L)	0·15	0·13	0·16	0·15	0·19	0·20	0·38^§^
BASO (X10^9^/L)	0·03	0·02	0·03	0·02	0·03	0·02	0·46^§^
NEUT %	58·56	7·60	59·79	8·51	58·61·	8·70	0·58^§^
LYMPH %	32·41	7·37	31·19	8·15	32·24·	8·27	0·60^§^
MONO %	6·08	1·51	5·97	1·57	5·91	1·28	0·85^§^
EO%	2·40	1·96	2·52	2·04	2·73	2·34	0·71^§^
BASO%	0·54	0·23	0·53	0·25	0·49	0·25	0·39^§^
PLT (X10^9^/L)	234·83	49·64	225·05	50·48	230·60	58·12	0·61^§^
NLR	1·94	0·62	2·12	0·88	2·03	0·98	0·55^§^
MLR	0·20	0·07	0·20	0·09	0·20	0·07	0·87^§^
PLR	128·18	32·54	132·63	43·70	129·08	56·34	0·84^§^
ESR (mm/h)	15·33	9·94	13·02	9·17	14·50	11·20	0·42^§^
CRP (mg/l)	2·25	2·00	1·80·	2·37	4·69·	14·12	0·08^§^
GLU (mmol/l)	5·97	1·54	5·67	1·14	5·83	2·05	0·60^§^
Ca^2+^ (mmol/l)	2·33	0·08	2·33	0·11	2·31	0·12	0·49^§^
Mg^2+^ (mmol/l)	0·88	0·06	0·86	0·06	0·86	0·09	0·39^§^
ALP (U/L)	91·43	23·78	85·95	20·80	82·36	18·75	0·11^§^
LDH (U/L)	201·20	29·45	198·06	33·84	203·11	34·29	0·58^§^
25(OH) (ng/ml)	16·26	3·00	25·56	2·80	35·83	5·67	< 0·001^§^
25(OH)D_2_ (ng/ml)	1·00	2·36	0·82	1·91	1·41	3·31	0·29^§^
25(OH)D_3_ (ng/ml)	15·27	3·40	24·73	3·15	34·43	6·10	< 0·001^§^

Vit-D, vitamin D; COPD, chronic obstructive pulmonary disease; CKD, chronic kidney disease; WBC, white blood cell count; NEUT, neutrophil count; LYMPH, lymphocyte count; MONO, monocyte count; EO, eosinophil count; BASO, basophil count; NEUT%, neutrophil ratio; LYMPH%, lymphocyte ratio; MONO%, monocyte ratio; EO%, eosinophil ratio; BASO%, basophil ratio; PLT, platelet; NLR, neutrophil:lymphocyte ratio; MLR: monocyte:lymphocyte ratio; PLR: platelet:lymphocyte ratio; ESR: erythrocyte sedimentation rate; CRP, C-reactive protein; GLU, glucose; ALP, alkaline phosphatase; LDH, lactate dehydrogenase.

*P*-values were determined using the Pearson *χ*^2^ test^**†**^, Fisher exact test^‡^ or ANOVA^§^.

### Effect of serum vitamin D concentrations on COVID-19 incidence, severity and reoccurrence rate

The COVID-19 incidence was higher in the vitamin D deficiency group compared with the insufficiency and sufficiency groups (83 % *v*. 69 % *v*. 58 %, *P* = 0·03). Furthermore, the vitamin D deficiency group also exhibited a higher reoccurrence rate of COVID-19 (28 %) compared with the insufficiency group (16 %) and sufficiency group (8·2 %) (*P* = 0·02). However, no significant difference was observed in COVID-19-related hospitalisation rates among the groups (*P* = 0·30). (Table 2)

**Table 2:** Comparison of incidence, hospitalisation and reoccurrence of COVID-19 among participants with different concentrations of vitamin D

	Deficiency (*n* 30)		Insufficiency (*n* 108)		Sufficiency (*n* 84)		
	*n*	%	*n*	%	*n*	%	*P*-value
Incidence	25	83	75	69	49	58	0·03
Hospitalisation	2·	8·0	2·	2·7	1·	2·0	0·30^*^
Reoccurrence	7	28	12	16	4·	8·2	0·02^*^

Reoccurrence: reoccurrence numbers/incidence.

* Fisher exact test.

**Fig. 3:**
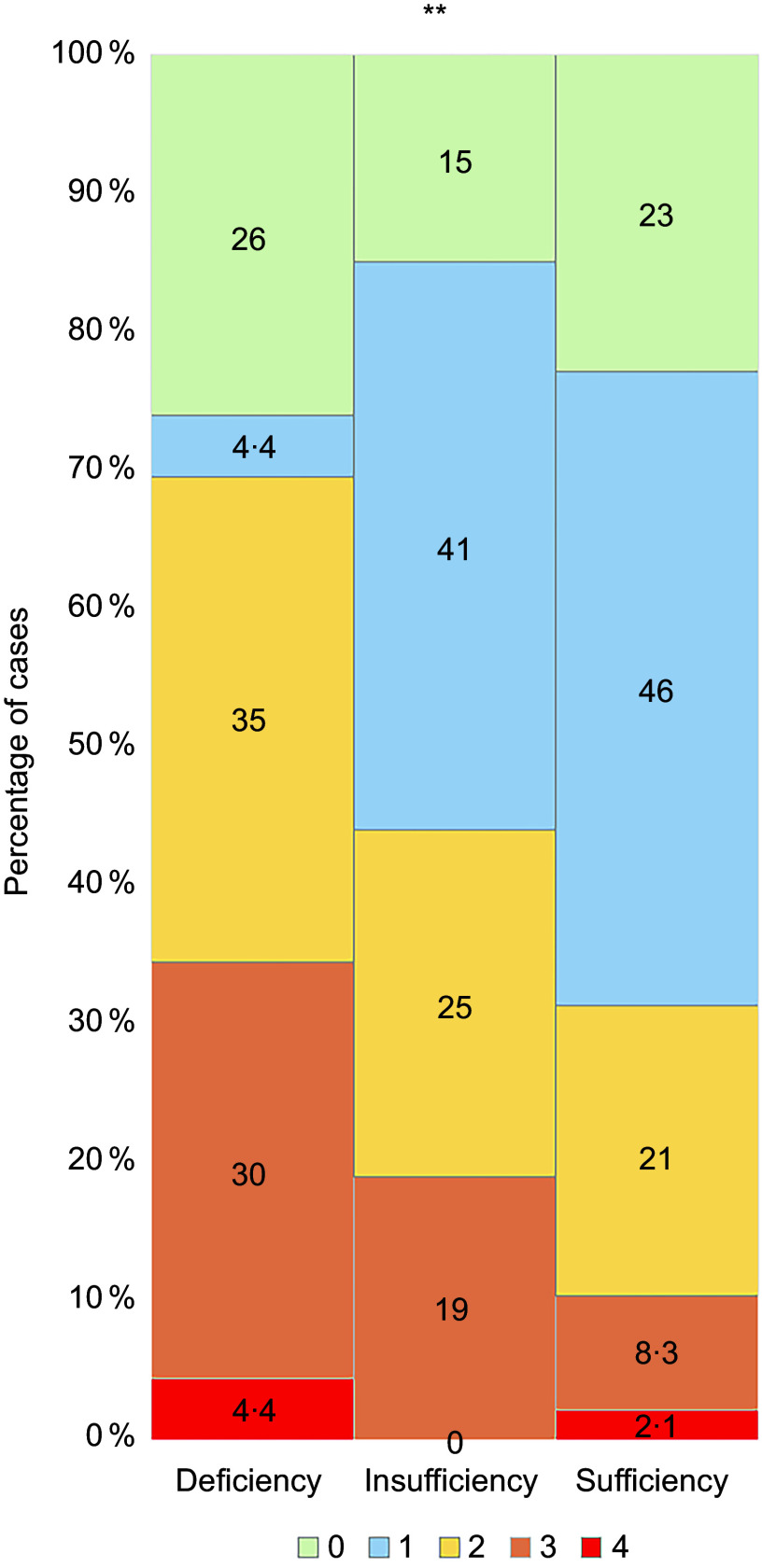
Comparison of severity of COVID-19 in vitamin D deficiency, insufficiency and sufficiency groups. (a) Mild cases: level 0–1. (b) Moderate case: level 2. (c) Severe case: level 3. (d) Critical case: level 4. Fisher’s exact test. **: *P* = 0·003

In addition, the three groups also differed in the severity scores of COVID-19 (*P* = 0·003). The proportion of mild cases (level 0 and level 1) was highest in the vitamin D sufficiency group (69 %) and lowest in the vitamin D deficiency group (30 %). In contrast, the severe and critical cases (level 3 and level 4) were higher in the vitamin D deficiency group (35 %) than the vitamin D insufficiency group (19 %) and vitamin D sufficiency group (10 %). (Fig. 3)

### Association between vitamin D supplementation and incidence and reoccurrence rate of COVID-19 using binary logistic regression analysis

The dependent variables were incidence (*v*. no incidence) or reoccurrence (*v*. no reoccurrence). Vitamin D supplementation was selected as the independent variable based on the results from ANOVA with a *P*-value < 0·05. Before data analysis, the assumption of multicollinearity was tested, and there was no collinearity. The statistical results showed that vitamin D supplementation was not statistically significant (*P* > 0·05). Thus, vitamin D supplementation was not a significant predictor of COVID-19 incidence and reoccurrence rate in the general population (Table 3).

**Table 3:** Binary logistic regression results of vitamin D supplementation correlates to the incidence and reoccurrence rate of COVID-19

Variable	*β*	se	*P*-value	OR	95 % CI
Incidence*					
Vitamin D supplementation (yes *v*. no)	0·10	0·37	0·78	0·90	0·54, 2·26
Constant	1·92	0·57	0·001	7·56	
Reoccurrence rate^†^					
Vitamin D supplementation (yes *v*. no)	0·07	0·51	0·89	1·08	0·40, 2·91
Constant	0·22	0·84	0·79	1·25	

*2 Log-likelihood = 274·72; Cox and Snell R^2^ = 0·03; Nagelkerke R^2^ = 0·04; Hosmer and Lemeshow test: x^2^ = 5·69, *P* = 0·68. ^†^2 Log-likelihood = 138·68; Cox and Snell R^2^ = 0·04; Nagelkerke R^2^ = 0·08; Hosmer and Lemeshow test: x^2^ = 2·47, *P* = 0·96. ^a^Variable(s) entered in step 1: vitamin D supplementation. 25(OH)D, 25(OH)D_3_.

### Ordinal logistic regression results of the relationship between vitamin D supplementation and COVID-19 severity

The five-level ordinal COVID-19 outcomes were the dependent variable, and vitamin D supplementation was the independent variable of interest. The predictor variable of this model was also selected according to the results of ANOVA (*P* < 0·05). The results of ordinal logistic regression analysis showed that vitamin D supplementation had no association with the severity of COVID-19 in individuals (*P* > 0·05) and was not considered a risk factor for the severity of disease (Table 4).

**Table 4:** Results of vitamin D supplementation correlates to the severity level of COVID-19: ordinal logistic regression model

Variable	*β*	se	*P*-value	OR	95 % CI
Severity level					
Vitamin D supplementation (no *v*. yes)	−0·40	0·36	0·27	0·67	−1·09, 0·30

2 Log-likelihood = 426·49; goodness of fit: Cox and Snell R^2^ = 0·03; Nagelkerke R^2^ = 0·03; McFadden R^2^ = 0·009; test of parallel lines: *P* = 0·49.

## Discussion

In this study, we observed a higher incidence rate of COVID-19 among individuals with vitamin D deficiency compared with those with vitamin D insufficiency or sufficiency. Moreover, individuals with vitamin D deficiency who contracted COVID-19 were more likely to experience severe/critical cases. Additionally, within the group of individuals who had contracted the virus, those with vitamin D deficiency had a significantly higher reoccurrence rate compared with those with vitamin D insufficiency or sufficiency.

Unlike previous studies that primarily focused on hospitalised COVID-19 patients, our study examined a cohort of elderly individuals who underwent routine medical examinations shortly before potentially contracting COVID-19. Most studies involving hospitalised COVID-19 patients have consistently reported an association between lower serum vitamin D concentrations and increased COVID-19 severity and mortality^(17,21,23,24)^. However, studies from Saudi Arabia and Kuwait did not find a significant association between serum 25(OH)D levels and disease severity or mortality in hospitalised COVID-19 patients^(15,16)^. It is important to note that in these studies, vitamin D concentrations were measured after patients were already infected, potentially influenced by the systemic inflammatory response induced by COVID-19 itself^(25)^. In contrast, our study focused on individuals aged 60 years and older, with a mean age of 68 years. In studies that did not support the relationship between vitamin D and COVID-19, younger patients were typically included, such as those aged 4–60 years^(15)^ or with a mean age of 49 ± 17 years^(16)^. To investigate the association between vitamin D levels and COVID-19 more effectively, it is reasonable to assess vitamin D concentrations in the general population just before infection, as we did in our study.

Prior research using data from the UK Biobank did not find a significant link between 25(OH)D levels and COVID-19 susceptibility, severity, hospitalisation or mortality^(26,27,38,39)^. However, it is worth noting that the 25(OH)D serum samples analysed in the UK Biobank studies were collected years before the patients’ infections and may not accurately represent their pre-infection vitamin D status. In contrast, a study of a diverse cohort of over 4599 veterans with positive COVID-19 tests found that serum 25(OH)D concentrations measured 15–90 d before testing were independently associated with COVID-19-related hospitalisations and mortality in an inverse dose–response relationship^(9)^. Our study aligns with this approach, as we measured pre-infection serum 25(OH)D concentrations in individuals who were not COVID-19 patients but rather local residents undergoing routine annual medical examinations.

Furthermore, our study was conducted during a period when the Omicron variant was responsible for a significant COVID-19 outbreak in late December 2022 and January 2023^(31,32)^. The infection rate was exceptionally high during this period, with approximately 89 % of the provincial population of nearly 100 million people infected^(30)^. Approximately 23 months after the first reported case of COVID-19, the Omicron variant was first reported in Africa in November 2021, and by early 2022, the Omicron variant was already the dominant strain worldwide, accounting for 99·7 % of the sequences registered from 23 February to 24 March 2022^(40)^. Therefore,iCOVID-19 mutant strains in the studies before November 2021 were COVID-19 mutant strains like alpha, beta and delta variants^(6–11,13–16)^. However, the effect of vitamin D levels on Omicron COVID-19 has not yet been reported.

Regarding vitamin D supplementation, we observed a higher percentage of individuals taking vitamin D supplements for at least 3 months in the vitamin D insufficiency and deficiency groups. However, binary and ordinal logistic regression models indicated that vitamin D supplementation was not significantly associated with COVID-19 severity in our study. Conflicting reports exist regarding the effectiveness of vitamin D supplementation in reducing the risk of COVID-19 infection and its severity. In a double-blind and parallel randomized controlled trial (RCT) study of highly exposed individuals, Villasis-Keever *et al*. found that the risk of SARS-CoV-2 infection was lower in those treated with vitamin D supplements with an elevated serum level of 25(OH)D^(41)^. While some studies have suggested a lower risk of infection with elevated serum 25(OH)D levels and a reduced risk of SARS-CoV-2 infection with habitual vitamin D supplement use^(39)^, others, such as the CORONAVIT trial, did not find a reduction in the risk of acute respiratory infections or COVID-19^(42)^. It is important to note that individuals who consume vitamin D supplements may not necessarily have significantly elevated vitamin D levels in circulation, as was the case in our study. Furthermore, the definition of a normal serum vitamin D concentration remains a subject of debate, with some arguing that a serum concentration of 30 ng/ml is the minimum for adequate immunity, while others suggest that a range of 40–60 ng/ml may be more functionally adequate^(43,44)^. In our study, the protective effect of vitamin D was based on serum vitamin D concentrations rather than the regular intake of vitamin D supplements.

Our study has several limitations, including a limited sample size, imprecise measurement of vitamin D supplement dosages, the frequency of vitamin D intake, the potential for variations in vitamin D concentrations within 3 months before major disease onset, overlapping periods of vitamin D supplementing and COVID-19 contraction and the fact that participants in routine health screenings may not fully represent the local community.

In conclusion, our study suggests that serum vitamin D levels shortly before the major Omicron COVID-19 outbreak were associated with the incidence, severity and reoccurrence rate of COVID-19 in the elderly population.
